# Risk factors associated with in-hospital mortality patients with COVID-19 in Saudi Arabia

**DOI:** 10.1371/journal.pone.0270062

**Published:** 2022-06-24

**Authors:** Mohammed Aljuaid, Hadil Alotair, Farrah Alnajjar, Wadi Alonazi, Hanaa Sharaf, Eman Sheshah, Lolwah Alashgar, Mashael Alshaikh

**Affiliations:** 1 Department of Health Administration, College of Business Administration, King Saud University, Riyadh, Saudi Arabia; 2 Department of Medicine, King Saud University Medical City, King Saud University, Riyadh, Saudi Arabia; 3 Yanbu General Hospital, Ministry of Health, Riyadh, Saudi Arabia; 4 Department of Cytogenetics Laboratory, Ministry of Health, Riyadh, Saudi Arabia; 5 Department of Internal Medicine, King Salman Hospital, Ministry of Health, Riyadh, Saudi Arabia; 6 Department of Pharmacy Services, King Saud University Medical City, King Saud University, Riyadh, Saudi Arabia; Kaohsuing Medical University Hospital, TAIWAN

## Abstract

Risk factors for in-hospital mortality of COVID-19 patients in Saudi Arabia have not been well studied. Previous reports from other countries have highlighted the effect of age, gender, clinical presentation and health conditions on the outcome of COVID-19 patients. Saudi Arabia has a different epidemiological structure with a predominance of young population, which calls for separate study. The objective of this study is to assess the predictors of mortality among hospitalized patients with COVID-19 in Saudi Arabia. This is a retrospective observational cohort study of hospitalized adult COVID-19 patients at two tertiary hospitals in Saudi Arabia between May to July 2020. Electronic charts were retrospectively reviewed comparing survivors and non-survivors in terms of demographic and clinical variables and comorbid conditions. A total of 564 hospitalized patients with COVID-19 were included in the study. The overall in-hospital mortality rate was 20%. The non-survivors were significantly older than survivors (59.4 ± 13.7 years and 50.5 ± 13.9 years respectively P< 0.001). Diabetes mellitus, hypertension, heart failure and ischemic heart disease were more prevalent among non-survivors (P< 0.001). The mean values of glycosylated hemoglobin HgA1C, D-dimer, ferritin, lactate dehydrogenase LDH, Alanin aminotransferase ALT and creatinine were significantly higher among non-survivors (P < 0.05). Multivariate logistic regression analysis revealed that age (aOR = 1.04; 95% CI 1.02–1.08; P < 0.01), chronic kidney disease (aOR = 4.04; 95% CI 1.11–14.77; P < 0.05), acute respiratory distress syndrome ARDS (aOR = 14.53; 95% CI 5.42–38.69; P < 0.01), Mechanical Ventilation (aOR = 10.57; 95% CI 5.74–23.59; P < 0.01), Shock (aOR = 3.85; 95% CI 1.02–14.57; P < 0.05), admission to intensive care unit (ICU) (aOR = 0.12; 95% CI 0.04–0.33; P < 0.01) and length of stay (aOR = 0.96; 95% CI 0.93–0.99; P < 0.05) were significant contributors towards mortality. The in-hospital mortality rate of COVID-19 patients admitted to tertiary hospitals in Saudi Arabia is high. Older age, chronic kidney disease and ARDS were the most important predictors of mortality.

## Introduction

COVID-19 is currently the greatest challenge for the healthcare systems all over the world [1, 2]. Some argue that no specific treatment has been approved for this disease, apart from supportive care and prevention of complications. Despite this, there are now specific treatments like remdesivir, tocalizumab, steroids which have shown to decrease mortality. Most of the used treatments have been inferred from the experience of previous outbreaks with respiratory viruses [3, 4]. Therefore, the emphasis now is on the prevention of the infection and identification of vulnerable groups of patients who may have worse outcomes if infected.

Variable mortality rates have been reported from different countries across the world [2]. Many studies have identified different predictors of mortality including demographic features, clinical presentation and comorbidities [5–10]. A meta-analysis of 60 studies that included 51,225 patients from different hospitals in 13 countries had found a higher in-hospital mortality rate in older patients and smokers, and in those who present with dyspnoea and have kidney disease, hypertension, malignancy, diabetes and pulmonary disease [11].

Saudi Arabia has a different epidemiological structure with the predominance of the young population. The largest age group in Saudi Arabia is (35–39) year [12]. The last demographical survey by Saudi General Authority for statistics, showed that almost half of the population were aged between 25–54 years and that age group of 35–39 years was predominant [13]. In addition, the prevalence of Diabetes Mellitus in Saudi Arabia is one of the top 10 in the world [14]. Similarly, hypertension is highly prevalent among the Saudi population. In 2013, a national survey which was conducted on 10,735 Saudis aged 15 years or older, found that 15.2% and 40.6% of Saudis were hypertensive or borderline hypertensive, respectively [15].

It is, therefore, crucial to study the in-hospital mortality rate among patients with COVID-19 in the local population of Saudi Arabia and identify the predictors for worse outcome. This would be of great value not only for risk stratification of patients who warrant admission to hospitals but also to prioritize these patients for receiving COVID-19 vaccination. This study aimed to assess the potential risk factors associated with in-hospital mortality among patients with COVID-19.

## Materials and methods

This is a retrospective observational cohort study using the electronic medical records of hospitalized patients with COVID-19 from May to July 2020 at two tertiary hospitals in Riyadh, Saudi Arabia. The sample size comprised all patients aged between 18–80 years who had positive real-time reverse transcription-polymerase chain reaction (RT-PCR) for severe acute respiratory syndrome coronavirus 2 (SARS-CoV-2) in their nasopharyngeal swabs. Electronic charts were reviewed and compared between survivors and non-survivors in terms of demographic and clinical variables and comorbid conditions. Oncology patients and pregnant or lactating women were excluded from the study.

### Data collection

Demographic data about the age, gender, nationality, civil status, blood pressure, body mass index (BMI), history of smoking and comorbidities were included in this analysis. Symptoms and clinical presentation on the day of admission, medications, laboratory results were also collected for all subjects. The clinical outcomes included in-hospital mortality, length of hospital stay (including the intensive care unit (ICU) admission), pneumonia, acute respiratory distress syndrome (ARDS) as per Berlin definition [16, 17], mechanical ventilation, shock, acute kidney injury (defined as an abrupt (within 48 hours) reduction in kidney function based on an elevation in serum creatinine level, a reduction in urine output, the need for renal replacement therapy, or a combination of these factors) [18], and acute heart failure (defined as a rapid onset of new or worsening signs and symptoms of heart failure) [19]. All data were collected from COVID-19 patients’ electronic charts.

### Ethical approval

The study was approved by the KSUMC Institutional Review Board, reference number (Ref. No. 20/0497/IRB). This study was conducted in accordance with the Declaration of Helsinki. Patient consent to review their medical records was waived by the KSUMC IRB due to the anonymized data collection and maintained with confidentiality.

### Statistical analysis

Statistical analyses were performed using the Statistical Package for the Social Sciences software, version 25.0 (SPSS Inc., Chicago, IL, USA). Descriptive statistics were used to analyze the demographic and clinical variables for the baseline patient characteristics and outcomes. The categorical data were expressed as numbers and percentages, while for the continuous variables, mean ± standard deviation were presented. The categorical variables were analyzed using the Chi-square test, whereas the continuous data were compared using the independent sample t-test. The variables that showed significant differences in the univariate analysis were considered for analysis using multivariate logistic regression for establishing the association between the independent risk factors and mortality as the dependent variable. The results were articulated as adjusted odds ratio (aOR) and a 95% confidence interval (CI). P-value less than 0.05 was considered to be statistically significant.

## Results

### Baseline patient characteristics

The study included 564 patients with COVID-19 who were admitted to two tertiary hospitals in Riyadh, Saudi Arabia between May to July 2020. Their mean age was 52.3 ± 14.4 years, and 429 (76.1%) were men. One hundred and thirteen patients (20%) died during hospitalization and the majority of them (79.6%) were male. The non-survivors were significantly older than survivors (59.4 ± 13.7 years, 50.5 ± 13.9 years, respectively P< 0.001). Diabetes mellitus, hypertension, heart failure, chronic kidney disease, and ischemic heart disease were significantly more prevalent among non-survivors (P< 0.01). Blood pressure was significantly lower in non-survivors compared to survivors (P< 0.05). The majority of studied patients were married, however, survivors were more likely to be married than those who died (P< 0. 001). There was no difference among the two groups in terms of smoking and Body mass index and previous cerebrovascular accidents. Table 1 shows the baseline characteristics among survivors and non-survivors COVID-19 patients.

**Table 1:** Baseline characteristics of patients with COVID-19.

Variable	Total	Survivor	Non-Survivor	P-value
	(n = 564)	(n = 451)	(n = 113)	
Demographic data				
Age (years)	52.3± 14.4	50.5 ± 13.9	59.4 ± 13.7	< 0.001
Sex, male	429 (76.1)	339 (75.2)	90 (79.6)	0.318
Nationality, Saudi	191 (33.9)	114 (31.9)	47 (41.6)	0.052
Civil Status, Married	419 (74.3)	356 (78.9)	63 (55.8)	< 0.001
SBP (mmHg)	124.1 ± 27	125.3 ± 25.0	119.4 ± 33.0	0.039
DBP (mmHg)	72.8 ± 16.9	74.4 ± 15.8	66.7 ± 19.9	< 0.001
Body mass index (kg/m2)	29.6 ± 6.5	29.7 ± 6.5	29.5 ± 6.6	0.863
Smoker (vs non-smoker)	25 (4.4)	19 (4.2)	6 (5.3)	0.612
Comorbidities				
Diabetes	254 (45.0)	190 (42.1)	64(56.6)	0.006
Hypertension	202 (35.8)	142 (31.5)	60 (53.1)	< 0.001
Heart Failure	26 (4.6)	13 (2.9)	13 (11.5)	< 0.001
Chronic Kidney Disease	32 (5.7)	19 (4.2)	13 (11.5)	0.003
Chronic lung Disease	31 (5.5)	27 (6.0)	4 (3.5)	0.307
Ischemic Heart Disease	39 (6.9)	23 (5.1)	16 (14.2)	0.001
Cerebrovascular accident	25 (4.4)	17 (3.8)	8 (7.1)	0.126

Data are presented as mean ± standard deviation or number (%); DBP, Diastolic Blood Pressure; SBP, Systolic Blood Pressure.

The most common symptoms for the patients were shortness of breath (84.8%) followed by fever (79.3%) and cough (77.3%). However, there were no significant differences between the survivors and non-survivors as far as the symptoms were considered. Among medications, higher percentage of survivors received steroids, hydroxychloroquine and azithromycin than non-survivors (P< 0.01) and a small number of patients were receiving angiotensin-converting enzyme (ACE)-Inhibitors more in the non-survivor’s group.

Comparison of laboratory results showed that COVID-19 patients who did not survive had significantly higher mean values of glycosylated hemoglobin (HgA1C), D-dimer, low-density lipoprotein cholesterol (LDL), ferritin, lactate dehydrogenase (LDH), alanine aminotransferase (ALT) and creatinine (P< 0.05). The symptoms, medications and laboratory results of patients with COVID-19 are shown in Table 2.

**Table 2:** The symptoms, medications, laboratory results and outcomes among survivors and non-survivors of patients with COVID-19.

Variable	Total	Survivor	Non-Survivor	P-value
	(n = 564)	(n = 451)	(n = 113)	
Symptoms				
Cough	436 (77.3)	357 (79.2)	79 (70.0)	0.051
Fever	447 (79.3)	364 (80.9)	83 (74.1)	0.111
Shortness of Breath	478 (84.8)	388 (86.2)	90 (81.1)	0.172
Sputum increase	43 (7.6)	31 (6.9)	12 (10.6)	0.180
Hemoptysis	3 (0.5)	2 (0.4)	1 (0.9)	0.564
Sore Throat	39 (6.9)	32 (7.1)	7 (6.2)	0.736
Neurological	30 (5.3)	23 (5.1)	7 (6.2)	0.643
Diarrhea	76 (13.5)	63 (14.0)	13 (11.6)	0.513
Nausea and Vomiting	75 (13.3)	61 (13.5)	14 (12.4)	0.750
Abdominal Pain	25 (4.4)	21 (4.7)	4 (3.5)	0.606
Medications				
ACE-Inhibitors	55 (9.8)	36 (8.0)	19 (16.8)	0.005
ARBs-Inhibitors	42 (7.4)	34 (7.5)	8 (7.1)	0.868
Steroids	445 (78.9)	379 (84.2)	66 (58.4)	< 0.001
Cephalosporins	533 (94.5)	429 (95.1)	104 (92.0)	0.198
Hydroxychloroquine	272 (48.2)	253 (56.1)	19 (16.8)	< 0.001
Azithromycin	441 (78.2)	379 (84.0)	62 (54.9)	< 0.001
Anti-viral	56 (9.9)	42 (9.3)	14 (12.4)	0.328
Laboratory Results				
HgA1C	8.2± 4.4	7.9 ± 2.3	9.7 ± 10.5	0.014
D-dimer	2.6± 3.5	2.2 ± 2.9	4.5 ± 5	< 0.001
Ferritin	837.4± 959.5	715.9 ± 701.6	1344.8 ± 1552.1	< 0.001
LDH	498.1± 234.5	464.1 ± 199.4	643.9 ± 308.1	< 0.001
ALT	67.2± 92.6	63.4 ± 77.9	82.7 ± 136.5	0.049
Creatinine	128.3± 157.8	111.1 ± 133.5	197.9 ± 218.9	< 0.001

Data are presented as mean ± standard deviation or number (%); LDL, low-density lipoprotein cholesterol; ALT, alanine aminotransferase; LDH, lactic dehydrogenase; ACE: angiotensin converting enzyme, ARB: angiotensin receptor blocker; Cephalosporins (ceftriaxone or cefuroxime).

The Clinical outcomes of COVID-19 survivors and non-survivors are presented in Table 3. More patients in the non-survivor group developed shock, acute kidney injury (AKI), acute respiratory distress syndrome (ARDS) and required mechanical ventilation than survivors (P< 0.001). Furthermore, admission to the intensive care unit (ICU) and the length of stay at the hospital were significantly higher among non-survivors (P< 0.001, P = 0.001) respectively.

**Table 3:** The clinical outcomes among survivors and non-survivors of patients with COVID-19.

Variable	Total	Survivor	Non-Survivor	P-value
	(n = 564)	(n = 451)	(n = 113)	
Outcomes				
Pneumonia	516 (91.5)	417 (92.5)	99 (87.6)	0.098
ARDS	64 (11.3)	17 (3.8)	47 (41.6)	< 0.001
Mechanical Ventilation	99 (17.6)	24 (5.3)	75 (67.0)	< 0.001
Shock	41 (7.3)	6 (1.3)	35 (31.0)	< 0.001
Acute Kidney Injury	113 (20)	67 (14.9)	46 (40.7)	< 0.001
Heart Failure	19 (3.4)	16 (3.5)	3 (2.7)	0.638
ICU admission	150 (26.6)	74 (16.4)	76 (67.3)	< 0.001
LOS (day)	12.9 ± 12.5	12.1 ± 11.8	16.4 ± 14.7	0.001

Data are presented as mean ± standard deviation or number (%)

ARDS, Acute Respiratory Distress Syndrome; ICU, Intensive care unit; LOS, length of stay

The results of univariate and multivariate logistic regression analysis of the risk factors are summarized in Table 4. It is observed that the age (aOR = 1.04; 95% CI 1.02–1.08; P < 0.01), Chronic Kidney Disease (aOR = 4.04; 95% CI 1.11–14.77; P < 0.05), ARDS (aOR = 14.53; 95% CI 5.42–38.69; P < 0.01), Mechanical Ventilation (aOR = 10.57; 95% CI 5.74–23.59; P < 0.01), Shock (aOR = 3.85; 95% CI 1.02–14.57; P < 0.05), admission to ICU (aOR = 0.12; 95% CI 0.04–0.33; P < 0.01) and length of stay (aOR = 0.96; 95% CI 0.93–0.99; P < 0.05) were associated with a higher risk of in-hospital death. The values of aOR being greater than unity, age, chronic kidney, ARDS, mechanical ventilation and shock provide greater likelihood of mortality whereas admission to ICU and increased length of stay reduces the likelihood of death.

**Table 4:** Univariate and multivariate logistic regression analysis of the risk factors associated with mortality among COVID-19 patients.

Predictors	Univariate analysis				Multivariate analysis			
	95% CI				95% CI			
	OR	Lower	Upper	P-value	aOR	Lower	Upper	P-value
Demographic data								
Age (years)	1.06	1.03	1.06	< 0.001	1.04	1.02	1.08	0.001
Comorbidities								
Chronic Kidney Disease	2.96	1.41	6.18	0.004	4.04	1.11	14.77	0.034
Diabetes	1.79	1.18	2.72	0.006	-	-	-	-
Heart Failure	4.38	1.97	9.74	< 0.001	-	-	-	-
Hypertension	2.46	1.62	3.75	< 0.001	-	-	-	-
Ischemic Heart Disease	3.07	1.56	6.03	0.001	-	-	-	-
Medications								
Azithromycin	0.23	0.15	0.36	< 0.001				
Hydroxychloroquine	0.16	0.09	0.26	< 0.001	-	-	-	-
Steroids	0.26	0.17	0.41	< 0.001	-	-	-	-
Outcomes								
Acute Kidney Injury	3.93	2.49	6.21	< 0.001	-	-	-	-
ARDS	18.18	9.86	33.53	< 0.001	14.53	5.42	38.96	< 0.001
ICU admission	0.09	0.06	0.15	< 0.001	0.12	0.04	0.33	< 0.001
LOS (day)	1.02	1.00	1.04	0.002	0.96	0.93	0.99	0.002
Mechanical Ventilation	36.06	20.41	63.72	< 0.001	10.57	4.74	23.59	< 0.001
Shock	33.28	13.55	81.76	< 0.001	3.85	1.02	14.57	0.047

OR, Odds Ratio; aOR, adjusted Odds Ratio; 95% CI, 95% Confidence Intervals.

ARDS, Acute Respiratory Distress; LOS, Length of Stay; ICU, Intensive Care Unit.

## Discussion

This study has found that the in-hospital mortality rate among the COVID-19 cohort was 20%. Older age and chronic kidney disease were the most important predictors of in-hospital mortality. The non-survivors were more likely to develop acute kidney injury, ARDS, require mechanical ventilation and get admitted to ICU than survivors. Our findings are consistent with a systematic review and meta-analysis by Tian et al who reported a mortality rate of 31.4% amongst Chinese patients and 21.0% for New Yorkers [5].

On the contrary, Sheshah et al. from Saudi Arabia reported a lower mortality rate of 10% among COVID-19 patients and it was highest among Saudi males (28.9%) [20]. Similarly, Abohamr et al. showed a significantly lower mortality rate (4.27% fatality ratio) in a single centre in Riyadh, Saudi Arabia [21]. This fluctuation in mortality rate was reported by Alyami et al who noted an increase in mortality rate starting from March 2020, reaching the peak rate of deaths on 1 April 2020 (0.417 per 100 patients), and followed by non-linear reduction until 26 April 2020 (0.020 per 100 patients) [22]. The increased mortality in this study correlates with high rate of COVID-19 cases in the country, in addition, the admitted patients were severe cases 91.5% had pneumonia. They were older (mean age 52.3 years) more obese (mean BMI was 29,6). A high percentage had comorbid conditions patients (45% Diabetes & 35,8% Hypertension).

Indeed, in the present study patients who died were significantly older than patients who survived, their mean age was 52.3± 14.4 years old. This is in agreement with a recent study that analyzed data of 178,568 COVID-19 deaths from a total population of 16 countries and found that the mortality rate of COVID-19 was 8.1 times higher (95%CI  =  7.7–8.5) among those aged 55 to 64 years, and more than 62 times higher incidence rate ratios (IRR) (IRR  =  62.1; 95%CI  =  59.7–64.7) among those aged 65 or older compared with individuals aged 54 years or younger [23]. This could be explained by changes associated with immunosenescence in which the function of innate immune cells in older people is impaired, and therefore the effectiveness of viral clearance is reduced, and a dysregulated immune response is initiated [24]. In addition, older individuals are more likely to have a higher number of comorbid conditions than younger ones. In the present study, we had found that diabetes mellitus, hypertension, heart failure, ischemic heart disease and chronic kidney disease were significantly more prevalent among patients who died. It is consistent with the study of Imam Z. et al who reported comorbidities as independent mortality predictors in a large cohort of COVID-19 patients [25]. Furthermore, a meta-analysis that included 1389 COVID-19 patient (19.7% with severe disease) have shown a significant association of chronic kidney disease with severe COVID-19 [26]. This is in agreement with our findings that chronic kidney disease was a predictor of mortality. Similarly, Guan WJ. et al from China found that 38.1% versus 15.7% of severe COVID-19 cases had chronic kidney disease [27]. Few studies have suggested an association of the severity of symptoms with mortality [28]. However, when we compared the dead and surviving COVID-19 patient, there was no significant difference between them in relation to the symptoms.

In the present study, the survivors were more likely to be treated with Azithromycin, Steroids and Hydroxychloroquine. A retrospective cohort study of 1438 patients hospitalized in New York, had found that treating hospitalized COVID-19 patients with hydroxychloroquine, azithromycin, or both was not associated with significantly lower in-hospital mortality [29]. While another Multi-center retrospective observational study included 2,541 patients had found that treatment with hydroxychloroquine alone and in combination with azithromycin was associated with a reduction in COVID-19 associated mortality [30]. The use of steroids and in particular dexamethasone in patients with moderate to severe COVID-19 who are hypoxic has been shown to improve the overall outcome of these patients and is currently considered standard of care [31].

There are some possible limitations in this study. The first is the retrospective design of the study. The second limitation concerns the study setting, which was conducted in tertiary centers, thus could contribute to the higher mortality rate as these centers receive a very sick subset of patients. Also, the reliability of the findings will increase with increased sample size. More variables related to demographic and social outcomes could be considered in future studies.

## Conclusions

The in-hospital mortality rate of COVID-19 patients remains high. Older age, diabetes, hypertension, chronic kidney disease and ischemic heart disease are more prevalent among non-survivors. Age and Chronic kidney disease were independent risk factors for in-hospital mortality. It is important to implement an efficient algorithm for risk stratification of patients who are admitted to hospitals and to prioritize cases for receiving COVID-19 vaccination. Further studies are needed on a larger sample size to evaluate other important predictors related to COVID-19 outcome.

## References

[1] World Health Orginaztion (WHO). Rolling updates on coronavirus disease (COVID-19) 2020: WHO; 2020 [Available from: https://www.who.int/emergencies/diseases/novel-coronavirus-2019/events-as-they-happen).

[2] WHO. Coronavirus Disease 2019 (COVID-19) Situation Report–141 2020 WHO; 2020 [Available from: https://www.who.int/emergencies/diseases/novel-coronavirus-2019/situation-reports.

[3] Wiersinga WJ, Rhodes A, Cheng AC, Peacock SJ, Prescott HC. Pathophysiology, transmission, diagnosis, and treatment of coronavirus disease 2019 (COVID-19): a review. Jama. 2020;324(8):782–93. doi: 10.1001/jama.2020.12839 32648899

[4] Bartoli A, Gabrielli F, Alicandro T, Nascimbeni F, Andreone P. COVID-19 treatment options: a difficult journey between failed attempts and experimental drugs. Internal and Emergency Medicine.1–28. doi: 10.1007/s11739-020-02569-9 33398609 PMC7781413

[5] Tian W, Jiang W, Yao J, Nicholson CJ, Li RH, Sigurslid HH, et al. Predictors of mortality in hospitalized COVID‐19 patients: A systematic review and meta‐analysis. Journal of Medical Virology. 2020. doi: 10.1002/jmv.26050 32441789 PMC7280666

[6] Mehraeen E, Karimi A, Barzegary A, Vahedi F, Afsahi AM, Dadras O, et al. Predictors of mortality in patients with COVID-19–a systematic review. European journal of integrative medicine. 2020:101226. doi: 10.1016/j.eujim.2020.101226 33101547 PMC7568488

[7] Bonow RO, Fonarow GC, O’Gara PT, Yancy CW. Association of coronavirus disease 2019 (COVID-19) with myocardial injury and mortality. JAMA cardiology. 2020. doi: 10.1001/jamacardio.2020.1105 32219362

[8] Guo T, Fan Y, Chen M, Wu X, Zhang L, He T, et al. Cardiovascular implications of fatal outcomes of patients with coronavirus disease 2019 (COVID-19). JAMA cardiology. 2020. 10.1001/jamacardio.2020.1017 PMC7101506 32219356

[9] Kanne JP. Chest CT findings in 2019 novel coronavirus (2019-nCoV) infections from Wuhan, China: key points for the radiologist. Radiological Society of North America; 2020. doi: 10.1148/radiol.2020200241 32017662 PMC7233362

[10] Angelidi AM, Belanger MJ, Mantzoros CS. Commentary: COVID-19 and diabetes mellitus: What we know, how our patients should be treated now, and what should happen next. Metabolism. 2020;107:154245. doi: 10.1016/j.metabol.2020.154245 32320742 PMC7167295

[11] Mesas AE, Cavero-Redondo I, Álvarez-Bueno C, Sarriá Cabrera MA, Maffei de Andrade S, Sequí-Dominguez I, et al. Predictors of in-hospital COVID-19 mortality: A comprehensive systematic review and meta-analysis exploring differences by age, sex and health conditions. PloS one. 2020;15(11):e0241742. doi: 10.1371/journal.pone.0241742 33141836 PMC7608886

[12] General Authority for Statistics. Demography in Saudi Arabia. 2020. Available from: https://www.stats.gov.sa/sites/default/files/Population%20by%20Age%20Groups%20%2Cand%20Gender_0.pdf

[13] General Authority for Statistics. General Authority for Statistics Demographic Survey. Population by Age Groups, and Gender mid year 2019. Available from: https://www.stats.gov.sa/sites/default/files/population_by_age_groups_and_gender_ar.pdf

[14] Alwin Robert A, Abdulaziz Al Dawish M, Braham R, Ali Musallam M, Abdullah Al Hayek A, Hazza Al Kahtany N. Type 2 diabetes mellitus in Saudi Arabia: major challenges and possible solutions. Current diabetes reviews. 2017;13(1):59–64. doi: 10.2174/1573399812666160126142605 26813972

[15] El Bcheraoui C, Memish ZA, Tuffaha M, Daoud F, Robinson M, Jaber S, et al., Hypertension and its associated risk factors in the Kingdom of Saudi Arabia, 2013: a national survey. International journal of hypertension. 2014 Aug 6;2014. doi: 10.1155/2014/564679 25170423 PMC4142152

[16] McCormack V, Tolhurst-Cleaver S. Acute respiratory distress syndrome. Bja Education. 2017;17(5):161–5.

[17] Force ADT, Ranieri V, Rubenfeld G, Thompson B, Ferguson N, Caldwell E. Acute respiratory distress syndrome. Jama. 2012;307(23):2526–33. doi: 10.1001/jama.2012.5669 22797452

[18] Mercado MG, Smith DK, Guard EL. Acute kidney injury: diagnosis and management. American family physician. 2019;100(11):687–94. 31790176

[19] Kurmani S, Squire I. Acute heart failure: definition, classification and epidemiology. Current heart failure reports. 2017;14(5):385–92. doi: 10.1007/s11897-017-0351-y 28785969 PMC5597697

[20] Sheshah E, Sabico S, Albakr RM, Sultan AA, Alghamdi KS, Al Madani K, et al. Prevalence of Diabetes, Management and Outcomes among Covid-19 Adult Patients Admitted in a Specialized Tertiary Hospital in Riyadh, Saudi Arabia. Diabetes Research and Clinical Practice. 2020:108538. doi: 10.1016/j.diabres.2020.108538 33189790 PMC7661919

[21] Abohamr SI, Abazid RM, Aldossari MA, Amer HA, Badhawi OS, Aljunaidi OM, et al. Clinical characteristics and in-hospital mortality of COVID-19 adult patients in Saudi Arabia. Saudi Medical Journal. 2020;41(11):1217–26. doi: 10.15537/smj.2020.11.25495 33130842 PMC7804238

[22] Alyami MH, Naser AY, Orabi MA, Alwafi H, Alyami HS. Epidemiology of COVID-19 in the Kingdom of Saudi Arabia: An Ecological Study. Frontiers in public health. 2020;8. 10.3389/fpubh.2020.00506 PMC7527602 33042946

[23] Yanez ND, Weiss NS, Romand J-A, Treggiari MM. COVID-19 mortality risk for older men and women. BMC Public Health. 2020;20(1):1–7. 33213391 10.1186/s12889-020-09826-8 PMC7675386

[24] Kang SJ, Jung SI. Age-related morbidity and mortality among patients with COVID-19. Infection & chemotherapy. 2020 Jun;52(2):154. doi: 10.3947/ic.2020.52.2.154 32537961 PMC7335648

[25] Imam Z, Odish F, Gill I, O’Connor D, Armstrong J, Vanood A, et al.,. Older age and comorbidity are independent mortality predictors in a large cohort of 1305 COVID‐19 patients in Michigan, United States. Journal of internal medicine. 2020 Oct;288(4):469–76. doi: 10.1111/joim.13119 32498135 PMC7300881

[26] Henry BM, Lippi G. Chronic kidney disease is associated with severe coronavirus disease 2019 (COVID-19) infection. International urology and nephrology. 2020 Jun;52(6):1193–4. doi: 10.1007/s11255-020-02451-9 32222883 PMC7103107

[27] Guan WJ, Liang WH, Zhao Y, Liang HR, Chen ZS, Li YM, et al., Comorbidity and its impact on 1590 patients with COVID-19 in China: a nationwide analysis. European Respiratory Journal. 2020 May 1;55(5). doi: 10.1183/13993003.00547-2020 32217650 PMC7098485

[28] Islam MS, Barek MA, Aziz MA, Aka TD, Jakaria M. Association of age, sex, comorbidities, and clinical symptoms with the severity and mortality of COVID-19 cases: a meta-analysis with 85 studies and 67299 cases. medRxiv 2020. Preprint available at medRXiv doi: 10.1101/2020.05.23.20110965 PMC7737518 33344791

[29] Rosenberg ES, Dufort EM, Udo T, Wilberschied LA, Kumar J, Tesoriero J, et al., Association of treatment with hydroxychloroquine or azithromycin with in-hospital mortality in patients with COVID-19 in New York State. Jama. 2020 Jun 23;323(24):2493–502. doi: 10.1001/jama.2020.8630 32392282 PMC7215635

[30] Arshad S, Kilgore P, Chaudhry ZS, Jacobsen G, Wang DD, Huitsing K, et al., Treatment with hydroxychloroquine, azithromycin, and combination in patients hospitalized with COVID-19. International journal of infectious diseases. 2020 Aug 1;97:396–403. doi: 10.1016/j.ijid.2020.06.099 32623082 PMC7330574

[31] Wootton D. Dexamethasone in hospitalized patients with COVID-19. New England Journal of Medicine. 2021 Feb 25;384(8):693–704. doi: 10.1056/NEJMoa2021436 32678530 PMC7383595

